# A rare case of radius hemimelia: a case report

**DOI:** 10.11604/pamj.2022.41.304.32909

**Published:** 2022-04-14

**Authors:** Tejal Kishor Babar, Om Chandrakant Wadhokar, Mitushi Kishorrao Deshmukh

**Affiliations:** 1 Department of Musculoskeletal Physiotherapy, Ravi Nair Physiotherapy College, Datta Meghe Institute of Medical Sciences, Sawangi, Wardha, Maharashtra, India

**Keywords:** Hypoplastic radius, hemimelia, physical therapy, osteotomy, case report

## Abstract

Radial dysplasia congenital defect resulting in shortening of the forearm due to congenital shortening of the radius. Isidore Geoffroy Saint-Hilaire coined the term “hemimelia” around 1836-1837. Affected individuals may also have reduced limb functions abnormalities of the soft tissues, vasculature of the forearm. The management consist of splinting, stretching, and centralization. Physical therapy management plays a vital role in regaining hand function and improving quality of life. In severe cases, surgical correction such as osteotomy. Radial hemimelia is a rare disorder with 1/5000-30,000 live birth. A 16-year-old girl was admitted to Acharya Vinoba Bhave Rural Hospital (AVBRH) with complaints of weakness of the right upper limb along with a tingling sensation from the past 1 year. She was operated on with ulnar osteotomy and physical therapy management was initiated which consists of regaining mobility and strength and making the patient functionally independent. We concluded that a well structure physical therapy protocol along with medical therapy post-surgery improved the overall status of the patient.

## Introduction

A failure in the development of components that make up the radial side of the forearm characterizes radial longitudinal deficiency, a rare skeletal abnormality. This is accompanied by congenital dislocation of the dysplastic radial head [1]. Hemimelia is a congenital disorder in which one or more bones are missing completely or partially. The most widely accepted theory is that radial agenesis is caused by neural crest damage. Treatment options are determined by the severity of the deformity and the loss of limb function [2]. Other skeletal abnormalities associated with ulnar hemimelia include humeroradial synostosis, radial head dislocation, carpal or metacarpal coalition, and digital abnormalities [3]. According to Bunnell (1944), radial hemimelia is a deformity of the radial bud, which “frequently involves the carpal and digital rays of the radius, as well as the radial section of the end of the humerus [4]. Early detection is critical to minimize muscle atrophy and the development of bone abnormalities, which can hurt the patient's quality of life [5].

Clinical signs and radiographic examination revealed the total or partial absence of the afflicted bone as well as angular abnormalities, which are used to make the diagnosis [6]. This malformation is thought to appear between the 28^th^ and 56^th^ week of pregnancy. It is classified into the following types: Type I as short distal radius, Type II as hypoplastic radius, Type III as partial radius absence and type IV as complete radius absence [7]. Amelia, ectrodactyly, polydactyly, and syndactyly are all examples of hemimelia, which is congenital bone dysmorphology [2]. This abnormality is considered sporadic in humans [8], or extremely rare, given that the human incidence is about 1 in every 1 million live births [9]. We present a case of a 16 years old girl with complaints of weakness of the right upper limb along with tingling sensation from the past 1 year and was admitted to AVBRH for further Surgical and Physiotherapy management.

## Patient and observation

A 16-year-old girl was admitted to AVBRH with complaints of weakness of the right upper limb along with a tingling sensation from the past one 1 year. The patient had a past medical history of perinatal infection at the time of birth due to non-development of radius.

**Clinical findings:** the patient was assessed in the supine lying position. The patient was vitally stable. On observation, the patient had an ectomorphic build. The patient had an above-elbow cast over the right upper limb. On palpation, grade 2 tenderness was present at the site of operation with no evidence of swelling. The pain was graded 6/10 on Numerical Pain Rating Scale (NPRS). On examination the range of motion of the left upper limbs and bilateral lower limbs were full and functional. The ranges of motion of the right shoulder and wrist were full and functional ranges and strength of the elbow cannot be assessed due to the case.

**Timeline:** a 16-year-old girl with abnormal shortening of the right forearm was first noticed in 2007, diagnosis was done using an X-ray on 25/11/2021, for which ulnar osteotomy was suggested and done on 30/11/2021, and referred to physiotherapy on 02/12/2021.

### Diagnostic assessment

**Figure 1:**
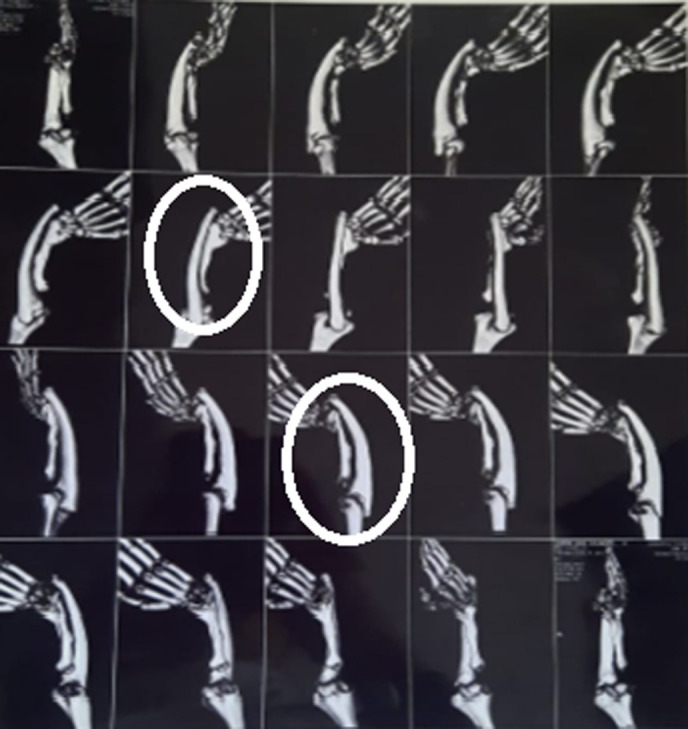
right hypoplastic radius

**Diagnostic methods:** the computerized tomography (CT) scan of the right forearm divulged evidence of non-visualization of proximal third of radius, with the shaft of ulna appearing curved and poorly supporting the wrist joint (Figure 1).

**Diagnostic challenges:** disease rarity, lack of etiology understanding, and lack of biomarkers are the few diagnostic challenges.

**Figure 2:**
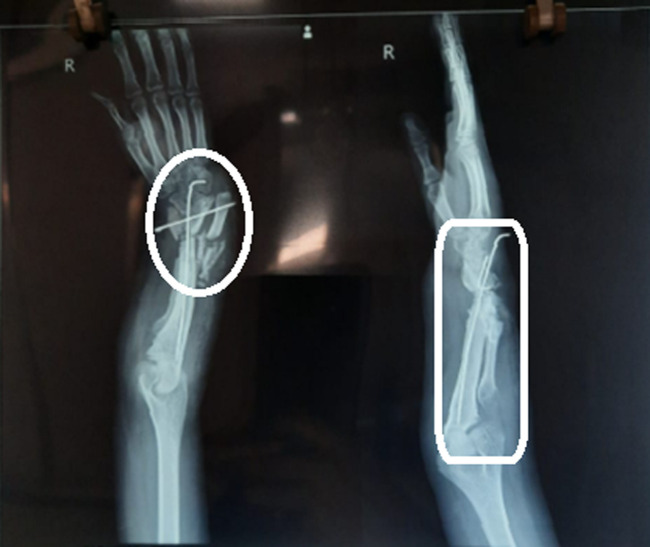
X-ray of the right side (upper limb)

**Diagnosis:** the final impression divulged radial ray anomaly Type II i.e Type II radial hemimelia (Figure 2).

**Physiotherapy management:** the patient and relatives were educated about the importance of physical therapy on a better and fast recovery. Electrotherapy modalities like ultrasound will be used in the later stages for aiding in the healing. Manual therapy techniques involving mobilization will be incorporated for maintaining joint integrity and improving joint range of motion. Detailed physical Therapy management is mentioned in Table 1.

**Table 1:** physiotherapy management

Intervention	Dosage	Rationale
Prehension activities (tip-tip, pad to pad, opposition)	10 repetitions	To maintain hand dexterity
Stretching exercises for forearm flexors and extensors)	3 repetitions with 30 sec	To maintain and improve tissue extensibility and avoid adhesion formation and improve range of motion
Strengthening exercises for bilateral lower limb, and upper limb	10 repetitions of each movement with 1kg weight cuff and a small rubber bad for strengthening of finger flexors and extensors	Prevent atrophy and weakness due to hospitalization
Breathing exercises	Deep inspiration through the nose with 4 seconds hold followed by slow expiration through the mouth	To improve lung compliance and prevent accumulation of secretion

**Follow-up and outcome measures of interventions:** after 4 weeks, the patient´s follow-up was taken and the values were as follows: 1) Numerical Pain Rating Scale (NPRS); pre-treatment NPRS score: 6/10, post-treatment NPRS score: 2/10. 2) The Disabilities of the Arm, Shoulder, and Hand (DASH) score; pre-treatment: 81.0, post-treatment: 50.8.

**Patient perspective:** after successful surgery we have initiated physical therapy and medical therapy, a well-planned physical therapy was initiated twice daily which have improved the mobility of the elbow, wrist and hand and ease in activities of daily living activities such as toileting and grooming.

**Informed consent:** the patient and relatives were informed about the case report and oral consent was obtained.

## Discussion

The results of a study of 200 congenital limb deficit patients who visited the artificial limb centre in pune, India between January 1984 and April 1990 are given. This group is typical of the country's congenital limb deficiency population. In the upper limbs, the most common deficiencies were transverse phalangeal total/partial deficiency and transverse forearm partial deficiency (below elbow), while in the lower limbs, the most common deficiencies were transverse metatarsal total/partial deficiency and transverse leg partial deficiency (below the knee). Female children were more likely to have transverse forearm partial deficiency, but male children were more likely to have transverse leg partial deficiency. Sixteen (16) cases did not require therapy, while 6 simply required surgical correction. Thirty patients required surgery before receiving prosthetics, whereas 148 patients simply required prostheses. Approximately 68 percent of patients had good to outstanding recovery, whereas 18 percent had unsatisfactory rehabilitation. Although no conclusive explanation for the malformations could be identified, many parents suspected that probable eclipse exposure was to blame [10]. Another article describes a Gollop-Wolfgang complex instance lacking hand ectrodactyly.

A 5-year-old boy was admitted to the hospital with bilateral tibial hemimelia and a left femoral bifurcation. Because the patient's left limb lacked the knee extensor mechanism, disarticulation was performed. Tibiofibular synostosis was used to treat the right leg, which had Jones Type 2 tibia hemimelia. The patient is currently ambulant, with a prosthesis on the left leg and an ankle-foot orthosis on the right. The knee should be disarticulated in the absence of proximal tibial anlage, especially in individuals with femoral bifurcation. In the presence of a proximal tibial anlage with good quadriceps function, tibiofibular synostosis is a favorable option. Bifid femur with tibia hemimelia is a difficult congenital condition whose treatment is unknown. For Jones Type 2 tibia hemimelia, the authors prescribe tibiofibular synostosis and knee disarticulation for Type 1. Larger research, however, is required to bolster the recommendation (Table 2) [11].

**Table 2:** probable causative factors for congenital limb deficiencies

Causative factors (probable)	No. of patients
Antiemetics, antispasmodic and antibiotic drugs	19
History of miscarriage	8
Previous preterm births	3
Previous lower segment cesarian section	4
Abdominal trauma during pregnancy	4
Radiation during pregnancy	4
Genetics	1
Exposure to eclipse	19
**Total**	**62**

## Conclusion

We conclude that the combined treatment approach that is; medical therapy and physical therapy after surgery showed a significant improvement in the quality of life and contributed to the early recovery of the patient.

## References

[1] Özdemir M, Kavak RP, Akdağ M, Güven S (2020). An unusual phenotype of radial longitudinal deficiency (radial hemimelia) presenting in a young adult male. Radiol Case Rep.

[2] Pisoni L, Cinti F, Del Magno S, Joechler M (2012). Bilateral radial hemimelia and multiple malformations in a kitten. J Feline Med Surg.

[3] Özdemir M, Turan A, Kavak RP (2019). Ulnar hemimelia: a report of four cases. Skeletal Radiol.

[4] O´Rahilly R (1946). Radial hemimelia and the functional anatomy of the carpus. J Anat.

[5] Vilalta L, Franch J, Martorell J (2017). Radial hemimelia in a domestic rabbit (oryctolagus cuniculus). J Exot Pet Med.

[6] Makino H, Cruz TPPS da, Bezerra KS, Lima SR, Travagin DRP, Nespoli PB (2016). Partial unilateral radial hemimelia in feline. Acta Sci Vet.

[7] Radiopaedia Radial hemimelia.

[8] Corbera J, Pulido M, Morales M, Juste M, Gutiérrez C (2002). Radiological findings in three cases of paraxial radial hemimelia in goats. J Vet Med Sci Jpn Soc Vet Sci.

[9] Fernandez-Palazzi F, Bendahan J, Rivas S (1998). Congenital deficiency of the tibia: a report on 22 cases. J Pediatr Orthop Part B.

[10] Jain SK (1994). A study of 200 cases of congenital limb deficiencies. Prosthet Orthot Int.

[11] Ondari J, Kinyanjui J, Miano P, Sang E, Oburu E, Maru M (2018). Femoral bifurcation and bilateral tibial hemimelia: Case report. Pan Afr Med J.

